# Graduates of Lebanese medical schools in the United States: an observational study of international migration of physicians

**DOI:** 10.1186/1472-6963-7-49

**Published:** 2007-04-05

**Authors:** Elie A Akl, Nancy Maroun, Stella Major, Bechara Chahoud, Holger J Schünemann

**Affiliations:** 1 Department of Medicine, University at Buffalo, NY, USA; 2 Department of Social and Preventive Medicine, University at Buffalo, NY, USA; 3 Medical Education Teaching and Research Innovation Center (METRIC), University at Buffalo, NY, USA; 4 Department of Sociology, University at Buffalo, NY, USA; 5 Department of Family Medicine, American University of Beirut, Lebanon; 6 Department of Medicine, Lebanese University, Lebanon; 7 INFORMA, Italian National Cancer Institute Regina Elena, Rome, Italy

## Abstract

**Background:**

As healthcare systems around the world are facing increasing physician shortages, more physicians are migrating from low to high income countries. As an illustrative case of international migration of physicians, we evaluated the current number and historical trends of Lebanese medical graduates (LMG) in the US, and compared their characteristics to those of US medical graduates (USMG) and other international medical graduates (IMG).

**Methods:**

We evaluated the number of LMG using the 2004 the American Medical Association Physicians' Professional Data (AMA-PPD) and then compared it to the number of graduates of other countries. We evaluated the historical trends using the 1978–2004 historical files of the AMA-PPD. We analyzed the characteristics of all LMG and compared them to a random sample of 1000 USMG and a random sample of 1000 IMG using the 2004 AMA-PPD.

**Results:**

In 2004, there were 2,796 LMG in the US, constituting 1.3% of all IMG. Compared to other foreign countries contributing to the US physician workforce, Lebanon ranked 2nd after adjusting for country population size (about 4 million) and 21st overall. About 40% of those who graduated from Lebanese medical schools in the last 25 years are currently active physicians in the US. Since 1978, the number of LMG in the US showed a consistent upward trend at a rate of approximately 71 additional graduates per year. Compared with USMG and IMG, LMG were more likely to work in medical research (OR = 2.31; 95% Confidence Interval (CI) = 1.21; 4.43 and OR = 2.63; 95% CI = 1.34; 5.01, respectively) and to be board certified (OR = 1.43; 95% CI = 1.14; 1.78 and OR = 2.04; 95% CI = 1.65;2.53, respectively) and less likely to be in family practice (OR = 0.14; 95% CI = 0.10; 0.19 and OR = 0.18; 95% CI = 0.12; 0.26, respectively).

**Conclusion:**

Given the magnitude and historical trends of migration of LMG to the US, further exploration of its causes and impact is warranted. High income countries should consider the consequences of their human resources policies on both low income countries' and their own healthcare systems.

## Background

According to the World Health Organization's World Health Report (2000), human resources are "the most important of the health system's inputs" [1]. These resources are essential to combat health crises in some of the world's poorest countries and to build sustainable health systems in all countries [2]. As demands on these systems have been rapidly growing worldwide, demands on their human resources have also grown [3]. Many high income countries (HIC) responded to these demands by recruiting healthcare workers mainly from low income countries (LIC), resulting in a large wave of international migration, among others, of physicians [4,5].

The migration of Lebanese medical graduates (LMG) to the United States (US) is an illustrative example of the international migration of physicians. The US is a major receiving country where physicians are over-represented among the foreign-born compared to the total labor force [3]. Recent evidence shows that physician shortages in the US will probably worsen over the next 20 years [6]. The projected deficit by 2020 or 2025 could be as great as 200 000 physicians – 20% of the needed workforce [6]. The Council on Graduate Medical Education (COGME) in the US has called to increase the number of physicians, with international recruitment being one of the options [7]. Lebanon is a source country of physicians with the highest emigration factor in the Middle East and North Africa and the 7th highest in the World [8]. The emigration factor is an approximate percentage of medical school graduates from a source country working in one of four major recipient countries [8]. Lebanon, South Africa, India and Jordan's respective emigration factors are 19.3, 18.5, 10.6 and 6.4% [8].

In order to effectively manage the problems of international migration and of local shortages, there is a need to build an evidence base for the magnitude of the problem and the labor market contexts [3]. Such evidence base is missing for the case of migration of LMG to the US. The objective of this study was to evaluate the current number and historical trends of LMG in the US, to describe their characteristics and to compare them to the US medical graduates (USMG) and other international medical graduates (IMG) in the US.

## Methods

We conducted a cross sectional study using the American Medical Association Physicians' Professional Data (AMA-PPD) as our sampling frame [9]. This dataset contains detailed information on all physicians who reside in the United States and who have met the educational and credentialing requirements necessary for recognition as physicians [9]. A record is created when an individual enters a medical school accredited by the Liaison Committee on Medical Education (LCME), or in the case of IMG, upon entry into ACGME-accredited programs. Physicians are classified as active unless they are retired, semi-retired, working part-time, temporarily not in practice, or not active for other reasons. Active physicians include both physicians in practice and physicians in training who we will refer to as practicing physicians and residents respectively. We analyzed data from both current and historical files. The most current file available to us was the 2004 AMA-PPD.

First, we compared the number of LMG currently active in the US to that of graduates of other foreign countries before and after adjusting for country populations size [10]. We also obtained the number of those who graduated from Lebanese medical schools in the last 25 years and calculated the percentage of them currently active physicians in the US.

Second, we evaluated the historical trends of the number of LMG in the US using the historical files of the AMA-PPD spanning the 1978–2004 time period. We further evaluated the trend for the 2 subgroups of residents and practicing physicians. We used linear and polynomial models to depict the long term time trends of numbers and proportions for each of the three groups (LMG, USMG, and IMG) over the last quarter of century.

Third, using the 2004 AMA-PPD, we analyzed the characteristics of all LMG and compared them to a random sample of 1000 USMG and a random sample of 1000 IMG (not including Lebanese graduates). These characteristics included age, gender, years since graduation from medical school, practice specialty, practice type, practice location, primary employer and board certification. We described the practice location by linking its zip code to a four-category, rural-to-urban status and taxonomy, a condensed version of the Rural-Urban Commuting Area (RUCA) codes [11]. The RUCA codes represent a detailed and flexible scheme for delineating sub-county components of the US settlement system and are based on the 2000 decennial census [11]. We based the sample sizes of the USMG and IMG on a power calculation to detect a 5% difference between the groups in the percentage of board certification with a power of 0.80.

We conducted univariate analyses using the independent samples t-test and Chi-Square test where appropriate. In a first multivariable analysis, we used practice specialty as dependent variable and age, gender, country of graduation, and residency status (yes/no) as independent variables. We then conducted multivariable analyses using consecutively board certification, practice type, practice location, and primary employer as dependent variables and age, gender, country of graduation, residency status (yes/no), and practice specialty as independent variables. We excluded residents from analyses of board certification but otherwise adjusted for the same covariates in all models. We did not use the number of years since graduation as an independent variable in the main analyses because it was highly correlated with age (Pearson correlation coefficient = 0.95; p < 0.001). We however used it instead of age in secondary analyses to check the stability of the inferences. We used multinomial logistic regression models for all analyses except for that of board certification for which we used a binary logistic model. We considered two-sided p values and p < 0.05 as statistically significant. We used Microsoft Excel for data management and SPSS, version 13.0 (SPSS, Inc., Chicago, Illinois), for all analyses.

## Results

### Current number of LMG

In 2004, there were 2,710 LMG in the US constituting 0.3% of all active physicians (n = 910,755) and 1.3% of IMG (n = 215,928). Five hundred and five (18.6%) of LMG were residents. Based on unadjusted numbers, Lebanon ranked in the 21^st ^position among foreign countries from where physicians active in the US graduated (Table 1) [12]. After adjusting for country population size (about 4 million for Lebanon), Lebanon ranked in the 2^nd ^position after Grenada. 39.5% of those who graduated from Lebanese medical schools in the last 25 years are currently active physicians in the US.

**Table 1:** Ranking of foreign countries by the number of medical graduates they contribute to the US physician workforce, with and without adjusting for country population size.

Rank	Country ranking per number of graduates practicing in the US adjusted to country population	Country ranking per number of graduates practicing in the US (number of graduates)
1	Grenada	India (42,880)
2	Lebanon	Philippines (19,523)
3	Israel	Mexico (12,256)
4	Dominican Republic	Pakistan (10,224)
5	Cuba	Former USSR (5,343)
6	Philippines	Grenada (4,812)
7	Syria	Italy (4,805)
8	Taiwan	Egypt (4,791)
9	Mexico	South Korea (4,648)
10	Spain	Spain (4,367)
11	South Korea	China and Hong Kong (4,316)
12	Italy	Germany (4,240)
13	Colombia	Iran (4,220)
14	Pakistan	Dominican Republic (3,645)
15	Iran	Syria (3,366)
16	Egypt	England (3,042)
17	Germany	Israel (2,972)
18	England	Taiwan (2,833)
19	India	Colombia (2,817)
20	Former USSR	Cuba (2,817)
21	China and Hong Kong	Lebanon (2,710)

### Historical trends of LMG

**Figure 1:**
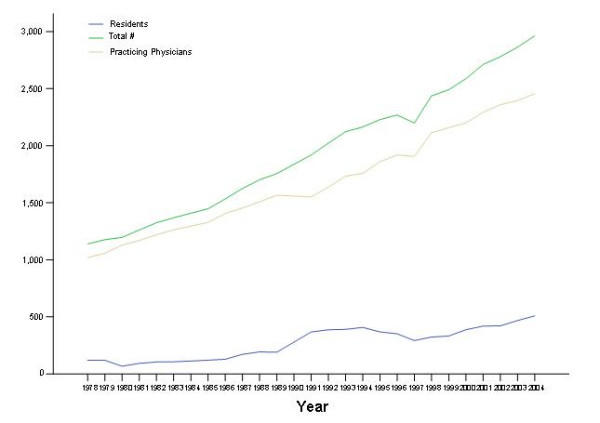
Time trends (1978–2004) of the number of Lebanese medical graduates in the US (number of residents, of practicing physicians and total number). Data for 1984 and 1990 are missing; we used straight interpolation in drawing the trends.

Between 1978 and 2004 the numbers of residents, practicing physicians and total LMG in the US showed a parallel and consistent upward trend (Figure 1). The upward trend for residents showed a "bump" in the early 1990's followed by a return to the baseline trend. The yearly number for each of the three groups (residents, practicing physicians, all LMG) fitted simple linear regression models and the addition of polynomial or logarithmic terms did not significantly improve curve estimation. The respective slopes and p values for the linear models were respectively: β = 16, p < 0.001; β = 55, p < 0.001; β = 71, p < 0.001. These results suggest that the total number of resident LMG, for example, increased by 16 each year.

### Characteristics of LMG, USMG and IMG

Table 2 displays the characteristics of LMG and compares them to those of USMG and IMG. Compared with both USMG and IMG, LMG were more likely to be residents, to work in medical research, to have a medical school or a non-governmental hospital as the primary employer, and to be board certified; they were younger, and less likely to be females, to have family practice or pediatrics as practice specialty, and to work in direct patient care or in a group practice. Compared with IMG, LMG had graduated more recently from medical school and were less likely to have family practice, internal medicine, or pediatrics as practice specialty or to have an HMO as a primary employer. Compared with USMG, LMG were more likely to practice in an urban location and less likely to practice in a large rural location.

**Table 2:** Comparison of the 2004 characteristics of the Lebanese (LMG), US (UMSGs) and International (IMG) medical graduates practicing in the US

		**LMG **(n = 2710)	**USMG **(n = 1000)	**IMG **(n = 1000)	**LMG vs. USMG**	**LMG vs. IMG**
		*Mean (sd)*	*Mean (sd)*	*Mean (sd)*	*P value*	*P value*

Age		45.4 (13.6)	47.6 (13.4)	50.5 (13.3)	<0.001	<0.001
Years since graduation		19.9 (13.3)	18.2 (13.0)	24.4 (13.0)	0.153	<0.001
						
		*%*	*%*	*%*	*P value*	*P value*

Gender	Female	17.3	26.1	30.0	<0.001	<0.001
Practice specialty^†^	Family Practice	1.8	12.1	8.3	<0.001	<0.001
	Internal Medicine	17.1	12.0	22.0		
	Pediatrics	5.9	7.5	8.7		
	OBGYN	5.0	5.5	4.1		
	General Surgery	5.5	4.6	3.1		
Practice type^§^	Resident	18.6	13.8	12.4	<0.001	<0.001
	Direct Patient Care	69.1	79.6	78.0		
	Administration	0.8	1.6	0.9		
	Medical Teaching	0.8	1.2	1.2		
	Medical Research	2.4	1.1	1.1		
Practice Location ‡	Urban	90.1	88.0	89.3	0.009	0.228
	Large Rural	4.2	6.8	5.6		
	Small Rural	1.8	2.2	1.6		
	Isolated Small Rural	0.5	0.8	0.8		
Primary Employer^¶^	Governmental	5.0	4.6	6.4	<0.001	<0.001
	Solo Practice	13.4	13.4	19.7		
	2 Physician Practice	2.6	3.3	2.6		
	Group Practice	19.6	31.2	21.1		
	HMO	0.0	0.3	0.5		
	Medical School	4.4	2.8	2.9		
	Non-Govt Hosp	23.0	17.2	16.2		
Board Certification^□^		86.0	81.1	72.5	.001	<0.001

The last two columns provide the p value for the difference of values for the variable of interest between two groups

^† ^Only primary care specialties are listed

^§ ^Only relevant categories are listed; data missing for n = 312

^‡ ^Data missing for n = 140

^¶ ^Only relevant categories are listed

^□ ^Residents excluded from denominator

The multivariable analyses confirmed that compared with USMG, LMG were more likely to work in medical research (OR = 2.31; 95% Confidence Interval (CI) = 1.21; 4.43) and to be board certified (OR = 1.43; 95% CI = 1.14; 1.78). LMG were less likely to be in family practice (OR = 0.14; 95% CI = 0.10; 0.19), or to work in a group practice (OR = 0.62; 95% CI = 0.48; 0.80). Compared with IMG, LMG were more likely to work in medical research (OR = 2.63; 95% CI = 1.34; 5.01), to have a medical school as the primary employer (OR = 2.16; 95% CI = 1.37; 3.41) and to be board certified (OR = 2.04; 95% CI = 1.65; 2.53). LMG were less likely to have family practice (OR = 0.18; 95% CI = 0.12; 0.26), internal medicine (OR = 0.47; 95% CI = 0.39; 0.58), or pediatrics (OR = 0.70; 95% CI = 0.53; 0.94) as practice specialty or to have an HMO as a primary employer (OR = 0.09; 95% CI = 0.01; 0.86). When we used years since graduation instead of age as an independent variable, the results did not change significantly except that, compared with USMG, LMG were more likely to have internal medicine as practice specialty (OR = 1.28; 95% CI = 1.01; 1.61) and to have a medical school as the primary employer (OR = 1.63; 95% CI = 1.01; 2.61).

## Discussion

Although LMG constituted only 1.3% of all IMG active in the US in 2004, Lebanon ranked second among the countries from where physicians in the US graduated after adjusting for country population size. 41.1% of LMG over the last 25 years are currently active physicians in the US. There has been a consistent upward trend in the number of LMG in the US since the late 1970s. Compared with USMG and IMG, LMG were more likely to work in medical research and to be board certified, and were less likely to be in family practice.

This study has a number of strengths. First, it is the first study attempting to build the evidence base for the migration of LMG to the US. Lebanon is arguably- relative to his population size – the foreign country that has contributed the most of its physicians to the US physician workforce (see below). Second, we used a dataset that is compiled using systematic and comprehensive methodology and accounts for all active physicians in the US[9] Third, we were able to assess the historical trends of this migration over a period of a quarter century reducing the possibility of chance findings that a simple cross-sectional analysis would bear. Fourth it accounts for the majority of LMG outside Lebanon. In fact, in 2004 there were only 17 LMG active in the United Kingdom (7 residents and 10 practicing physicians; personal communication, UK department of health) and 153 active in Canada (3 residents and 150 practicing physicians; personal communication, Canadian Medical Association). We could not obtain the numbers for France, the other country where LMG are known to be practicing medicine.

The study has some limitations. First, the AMA dataset does not include LMG who are in the US but not as licensed physicians (e.g. doing research exclusively). This number however should be very small. Second, in terms of its utility in investigating Lebanese human resources, this study looks at graduates of Lebanese medical schools and not at Lebanese physicians. In fact, it does not account for Lebanese physicians, graduates of non-Lebanese medical schools and active in the US. It also includes non-Lebanese physicians, graduates of Lebanese medical schools.

LMG differed from both USMG and IMG for most of the analyzed characteristics. Similar to our results, Freshnok et al. showed in the early 1980's that physicians in group practice tended to be younger and be US graduates[13]. Hagopian et al. compared the characteristics of Sub-Saharan African medical graduates to those of USMG[14]. As in the case of LMG, Sub-Saharan African graduates were younger, and more likely to be residents and work in urban locations [14].

One may argue that Lebanon ranks 1^st ^and not 2^nd ^among countries from where physicians active in the US graduated after adjustment for country size. In fact, less than 1% of Grenada graduates who are licensed in the US are Grenada citizens, while 82% of LMG in the US are Lebanese citizens [9]. In either case, the study findings indicate the seriousness of the Lebanese physicians' "brain drain" i.e. the loss of human capital and educational investment.

The historical trend data require a closer look. For example, the bump on the trend curve of residents in the early 1990s, the immediate post war period, is intriguing. Although the historical trend fitted a simple linear regression model one is tempted to relate it to a change in migration patters following the exacerbation of fighting in the late 1980s. Another possible explanation is dissatisfaction with the political, economic or social situation that followed the end of the civil war in 1990.

Many HIC including the US [15], the United Kingdom [16], Canada [17] and Australia [18] are planning to expand their physician workforce partly by recruiting from LIC. These countries should consider the consequences of such policies on both the sources countries' and their own healthcare systems [19]. While the source countries benefit from remittances and skills transfer,[20] they suffer from a brain drain, and losses in educational costs and returns from investment[21] In addition the healthcare systems of the source countries face reduced range of available services, and understaffing of facilities [22]. In many LIC, particularly in the Sub-Saharan Africa, the brain drain of physicians is a major impediment to disease-reduction initiatives [23,24]. It has even impacted some of these countries' ability to continuously develop academics to provide quality training of new doctors [25]. There is also indirect evidence that those who migrate might be the best among their peers [26]. The international recruitment of physicians can have consequences also for the HIC. In fact, with the increased threat of global pandemics, the public health in HIC increasingly depends on the effectiveness of healthcare systems in LIC [27].

The actions taken to deal the medical international recuritment of physicians are few and far between. A 2002 review identified eight codes of practice on ethical international recruitment of health professionals [28]. As an example, the UK Department of Health has published a Code of Practice "to promote high standards of practice in the international recruitment and employment of healthcare professionals" [29]. There is evidence however that the code has not affected the inflow of these professionals from "proscribed" countries [30].

The relative size and historical trends of migration of LMG to the US are remarkable. A qualitative study showed that the chief motivation for Lebanese medical students to train abroad was the need to gain a competitive advantage in an oversaturated Lebanese job market [31]. In fact, Lebanon suffers from an oversupply of physicians [32] and has a physician density of 325 physician per 100,000 (2001 data), the second highest in the Middle East and North Africa [33].

This study has important implications for research. There is a need to explore the causes of the oversupply of physicians in Lebanon. It is also important to study the impact of this migration on the Lebanese healthcare system. Finally, it would be interesting to explore whether the observed differences in practice characteristics (type, location and primary employer) among the studied groups reflect differences in personal preferences or in training and job opportunities.

## Conclusion

In managing the projected physician shortages policy makers in the US, and in HIC in general, should balance increasing the number of IMG with increasing the number of their own graduates. Lebanese policy makers should take an active role in managing the serious problem of physician brain drain [34].

## Competing interests

The author(s) declare that they have no competing interests.

## Authors' contributions

• Conception and design: EAA

• Collection and assembly of data: EAA, NM, SM, BC

• Analysis and interpretation of data: EAA, NM, HJS

• Drafting of the article: EAA

• Critical revision of the article for important intellectual content: EAA, NM, SM, BC, HJS

• Final approval of the article: EAA, NM, SM, BC, HJS

• Provision of study materials or patients: N/A

• Statistical expertise: EAA, HJS

• Obtaining of funding: EAA, HJS

All authors read and approved the final manuscript

## Pre-publication history

The pre-publication history for this paper can be accessed here:


